# Sarcopenia and coronary heart disease synergistically increase the risk of new onset depressive symptoms in older adults

**DOI:** 10.1186/s12877-021-02710-z

**Published:** 2021-12-24

**Authors:** Xiaoyu Chen, Peipei Han, Xing Yu, Yuanyuan Zhang, Peiyu Song, Yuewen Liu, Jinghuan Liu, Jiawei Tang, Yisong Zhang, Yong Zhao, Jiejiao Zheng, Lixi Chu, Hong Bing Wang, Qi Guo

**Affiliations:** 1 Department of Rehabilitation Medicine, Shanghai University of Medicine and Health Sciences Affiliated Zhoupu Hospital, 1500 Zhouyuan Road, Pudong New District, Shanghai, 201318 China; 2 Department of Rehabilitation Medicine, Tianjin Medical University, Tianjin, 300070 China; 3 Department of Rehabilitation Medicine, Shanghai Fourth Rehabilitation Hospital, Shanghai, 200040 China

**Keywords:** Coronary heart disease, Depressive symptoms, Elderly, Sarcopenia

## Abstract

**Background:**

Coronary heart disease (CHD), sarcopenia and depression are common disorders that markedly impair quality of life and impose a huge financial burden on society. They are also frequently comorbid, exacerbating condition and worsening prognosis. This study aimed to investigate the additive effects of CHD and sarcopenia on the risk of new onset depressive symptoms in older adults.

**Methods:**

The prospective cohort study comprised 897 Chinese community-dwelling participants who were aged 60 years and older (386 men; mean age 66.9 ± 5.9 years) without depressive symptoms at baseline, recruited from Chadian of Tianjin, China. Sarcopenia was defined according to the Asian Working Group for Sarcopenia (AWGS) criteria. CHD was identified via medical records or new diagnosed by at least two physicians. Depressive symptoms were assessed using the Geriatric Depression Scale (GDS) ≥11. Longitudinal data on new onset depressive symptoms were collected up to 12 months after baseline.

**Results:**

We found that 103 (11.5%) of the 897 participants without depressive symptoms at baseline had developed depressive symptoms. Participants were classified into mutually exclusive groups based on sarcopenia status and CHD: normal, CHD alone, sarcopenia alone, and co-occurring groups. A logistic regression showed that the CHD alone [odd ratios (OR) = 1.78, 95% confidence interval (CI) = 1.05–3.02], sarcopenia alone (OR = 2.79, 95% CI = 1.26–6.22), and co-occurring (OR = 7.19, 95% CI = 2.75–18.81) had higher risk of depressive symptoms than the normal group after adjusting for the covariates.

**Conclusions:**

CHD and sarcopenia synergistically increase the risk of new onset depressive symptoms in older adults. Thus, older adults may require early detection, and appropriate interventions should be implemented.

## Background

Depression is one of the most significant mental disorders associated with later life. A previous study reported that the prevalence of depressive symptoms among community-dwelling older adults varies from 8 to 16% [1]. Depression is a major risk factor for suicide, and is also related to considerable morbidity and mortality [2, 3]. Due to the severe side effects associated with depressive symptoms, researchers have sought to identify the risk factors for depression in elderly individuals and aim to reduce the incidence of depressive symptoms through intervention. Growing evidence supports that the circulatory and muscular systems are known to be associated with depression [4, 5]. Therefore, psychological and physical indicators need to be integrated together, so as to prevent the occurrence and development of diseases.

Coronary heart disease (CHD) is a psychosomatic disease with a prevalence of 19.3% in the older adults [6]. CHD and depression are also frequently comorbid, exacerbating the patient conditions and worsening prognosis. A recent review has reported that approximately 40% of people with CHD will also suffer from some form of depression, and as such are an important patient group, because they have worse physical health outcomes associated with CHD compared to similar patients without depression [7]. Meanwhile, accumulating evidence indicates that the onset of the episodes of depressive symptoms may be important in predicting worse outcomes among people with CHD [7, 8]. Accordingly, it should be expected that people with CHD at higher risk of new-onset depression would benefit from detection in enough time to prevent adverse outcomes. In addition, the direction of association between CHD and depressive symptoms is unclear, which needs further research. Therefore, a better understanding of comorbid CHD and depressive symptoms is particularly important for improving CHD management in older adults and thus achieving a healthier aging society.

It is noteworthy that sarcopenia is common in the elderly, with a prevalence in our previous study of nearly 10% [9]. Sarcopenia, defined as age-related loss of muscle mass and function [10], is a morbid condition in older adults that can result in serious health consequences, such as falls, disability and mortality [11]. Although the causal association between sarcopenia and depressive symptoms is still unclear, our previous cohort study showed that sarcopenia was an independent risk factor of depressive symptoms in suburb-dwelling older adults [12]. Furthermore, previous studies revealed that there may be an interaction relationship between sarcopenia and CHD [13–15]. CHD and sarcopenia often affect the same elderly individuals, seriously affecting the health and quality of life of the older adults. Thus, we have reason to believe that CHD and sarcopenia might be associated, accelerating the negative cycle of depressive symptoms through these interaction pathways. The high prevalence of these two factors makes the study of their joint effect very valuable. Inactivity may lead to CHD, meaning the physical decline of patients with CHD should be focused on to avoid the appearance of sarcopenia. When one risk has already emerged, we can control the other to prevent further complications. However, combinatory effects of these two conditions on depressive symptoms still remains unclear. Therefore, the aim of this study was to examine whether CHD and sarcopenia were independent risk factors, and whether they synergistically lead to an increase in new-onset depressive symptoms in Chinese community-dwelling older adults, using longitudinal cohort data.

## Methods

### Study participants

Our study population consisted of residents of the Hangu area of Tianjin, China, who were aged ≥60 years old and participated in China’s national free physical examination program. Participants gave full, informed written consent to take part in the study and ethical approval was approved by the Ethics Committee at our University, and the methods were carried out in accordance with the principles of the Declaration of Helsinki. In this study analysis, data collected in July 2015 and 2016 were used as the baseline, and new cases of depressive symptoms were follow-up in July 2016 and 2017.

**Fig. 1:**
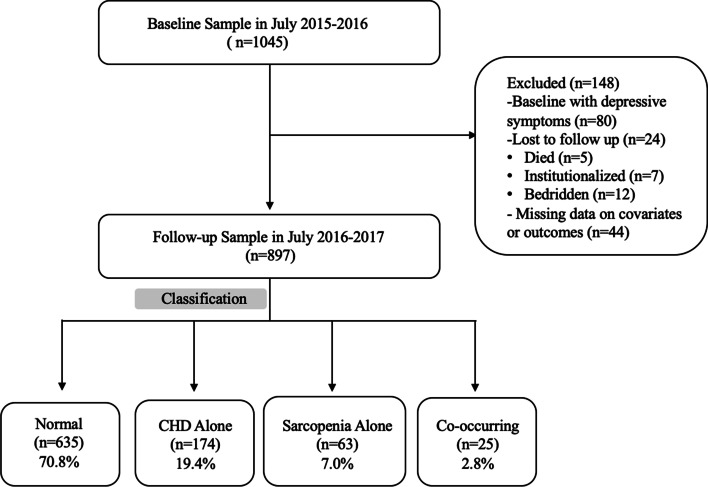
Flow diagram of the study

The inclusion criteria were volunteer to join in the study and absence of depressive symptoms at baseline. Exclusion criteria were as follows: (1) did not complete the assessment of depressive symptoms; (2) did not have the date of CHD diagnosis; (3) lack of relevant data for the assessment of sarcopenia or (4) cannot talk to interviewers or to grant informed consent. Baseline variable data were available for 1045 participants, although 80 older adults with depressive symptoms were excluded at baseline, 24 individuals were lost to follow-up (5 died, 7 institutionalized, 12 bedridden), and a further 44 had missing information for covariates or outcomes. Therefore, the final analytic date consisted of 897 participants (Fig. 1).

### CHD definitions

CHD history was obtained via medical records and reviewed by a cardiologist and a general practitioner, which included acute myocardial infarction, angina pectoris, percutaneous coronary intervention (PCI) or coronary artery bypass graft (CABG), and all other ischemic heart disease. In undiagnosed participants, at least two physician claims with a CHD diagnosis could be considered CHD [16].

### Assessment of sarcopenia and depressive symptoms

Sarcopenia was defined using the diagnostic algorithm according to the AWGS criteria [10] and the 30-item Geriatric Depression Scale (GDS) [17] was administered by conducting an interview to assess depressive symptoms [18]. Details of measurement methods can be found in our previous study [12].

### Covariates

Data related to sociodemographic variables (age, gender, marital status, educational level and occupation) and behavioral characteristics (smoking and drinking habits, sleep behavior, and history of falls). The short form of the International Physical Activity Questionnaire (IPAQ) [19] was used to evaluate the physical activity. We used the Mini Nutritional Assessment-Short Form (MNA-SF) [20], a validated screening tool used in geriatric health care, to evaluate the nutritional status, which had high sensitivity, specificity and correlation to the full MNA [21]. Comorbidity was assessed using the Charlson Comorbidity Index (CCI) [22] and current use of drugs included hypotensive drugs, hypoglycemic drugs, lipid-lowering drugs, cardiovascular drugs, psychotropic drugs, gastrointestinal drugs, or sleep drugs. Details of measurement methods have been described in our previous study [12] [23].

### Statistics

Participants were classified into mutually exclusive groups based on sarcopenia status and CHD status: normal, CHD alone, sarcopenia alone, and co-occurring. The study participants’ characteristics were compared by sarcopenia and CHD status using Student t-test or the Manne-Whitney U-test for continuous variables and the chi-square test for categorical variables. Using logistic regression analyses, we initially estimated odds ratio (OR) and 95% confidence intervals (CI) of sarcopenia alone, CHD alone, and both to assess the additive effects of sarcopenia and CHD on new onset depressive symptoms. Participants with no sarcopenia and no CHD were categorized as the reference group. The final model adjusted for age, sex, BMI, widowed, education, falling history, drinking, MNA-SF, sleep duration, sleep quality, hypotensive drugs, hypoglycemic drugs, cardiovascular drugs, sleep drugs and CCI. All statistical analyses were performed using SPSS v 25.0 (SPSS Inc., Chicago, IL), and *P* values of less than 0.05 were considered statistically significant.

## Results

Figure 1 shows the participants over the follow-up period. The analytic sample comprised 897 study participants (386 men; mean age at baseline 66.9 ± 5.9 years). At 1-year follow-up, there were 103 (11.5%) people with new onset elevated depressive symptoms. Demographics and clinical characteristics of the 4 groups are presented in Table 1.

**Table 1:** Baseline Characteristics of Study Participants absence of Depressive Symptoms at baseline

Variables	Normal	CHD Alone	Sarcopenia Alone	Co-occurring	*P*-value
	(*n* = 635)	(*n* = 174)	(*n* = 63)	(*n* = 25)	
Age (y)	66.0 ± 5.5	67.3 ± 5.6^a^	72.4 ± 6.5^a,b^	71.4 ± 6.3^a,b^	<0.001
Sex (Male)	314(49.4)	48(27.6)^a^	19(30.2)^a^	5(20.0)^a^	<0.001
BMI (kg/m^2^)	25.39 ± 3.30	26.02 ± 3.64^a^	22.68 ± 3.11^a,b^	22.51 ± 2.71^a,b^	<0.001
Grip strength (kg)	26.51 ± 9.53	23.32 ± 9.25^a^	16.04 ± 6.32^a,b^	16.52 ± 8.03^a,b^	<0.001
SMI (kg/m^2^)	7.31 ± 1.01	7.08 ± 1.09	5.43 ± 0.91^a,b^	5.43 ± 0.56^a,b^	<0.001
Gait speed (m/s)	1.01 ± 0.17	0.96 ± 0.16^a^	0.85 ± 0.19^a,b^	0.77 ± 0.22^a,b^	<0.001
IPAQ (Met/wk)	2142(924,4147)	1848(693,4186)	2079(1386,3066)	1038(0,4697)	0.732
Widowed (%)	76(12.0)	26(14.9)^a^	21(33.3)^b^	6(24.0)	0.001
Living alone (%)	90(14.2)	21(12.1)	16(25.4)	5(20.0)	0.253
Illiteracy (%)	152(24.0)	45(25.9)	28(44.4)^a^	7(28.0)	0.006
Farming (%)	559(88.2)	156(89.7)	57(90.5)	22(88.0)	0.911
Fall history (%)	84(13.2)	25(14.4)	12(19.0)	8(32.0)	0.044
Drinking (%)	90(14.2)	12(6.9)^a^	8(12.7)	0(0.0)	0.002
Smoking (%)	180(28.4)	47(27.2)	22(34.9)	10(40.0)	0.326
MNA-SF	13.06 ± 1.12	13.15 ± 0.90	12.19 ± 1.74^a,b^	12.08 ± 1.58^a,b^	< 0.001
Sleep Duration (h)	7.87 ± 1.32	8.12 ± 1.47	8.39 ± 1.30^a^	8.36 ± 2.33	0.005
Sleep quality (%)					0.003
Very well	339(53.4)	65(37.6)^a^	29(46.0)	7(28.0)	
Good	186(29.3)	61(35.3)	20(31.7)	9(36.0)	
Not enough	56(8.5)	20(11.6)	6(9.5)	6(24.0)	
Very poor	54(8.5)	27(15.6)	8(12.7)	3(12.0)	
Medicine use (%)					
Hypotensive drugs	222(35.0)	88(50.6)^a^	21(33.3)^b^	10(40.0)	< 0.001
Hypoglycemic drugs	41(6.5)	27(15.5)^a^	4(6.3)^b^	3(12.0)	< 0.001
Lipid-lowering drugs	78(12.3)	19(10.9)	2(3.2)	3(12.0)	0.237
Cardiovascular drugs	2(0.3)	102(58.6)^a^	3(4.8)^b^	11(44.0)^a,c^	< 0.001
Gastrointestinal drugs	16(2.5)	3(1.7)	3(4.8)	1(4.0)	0.382
Sleep drugs	34(5.4)	24(13.8)^a^	7(11.1)	5(20.0)^a^	<0.001
Chronic conditions (%)					
Diabetes	66(10.4)	30(17.2)	14(22.2)	6(24.0)^a,b^	0.003
Hypertension	320(50.4)	116(66.7)^a^	29(46.0)^b^	17(68.0)	< 0.001
Hyperlipidemia	236(37.2)	80(46.0)	11(17.5)^a,b^	11(44.0)	0.001
Stroke	36(5.7)	15(8.6)	6(9.5)	3(12.0)	0.257
Kidney disease	24(3.8)	10(5.7)	2(3.2)	1(4.0)	0.684
Hepatic disease	11(1.7)	2(1.1)	0(0.0)	2(8.0)	0.059
Biliary tract disease	19(3.0)	10(5.7)	4(6.3)	2(8.0)	0.164
Peptic ulcer	22(3.5)	12(6.9)	4(6.3)	2(8.0)	0.160
Osteoarthritis	82(12.9)	33(19.0)	8(12.7)	4(16.0)	0.231
Parkinson disease	2(0.3)	1(0.6)	0(0.0)	1(4.0)	0.316
Gout	3(0.5)	2(1.1)	0(0.0)	0(0.0)	0.642
CCI	2.45 ± 0.76	3.63 ± 0.76^a^	3.24 ± 0.88^a,b^	4.32 ± 1.31^a,b,c^	< 0.001

Notes: *CHD* coronary heart disease; *BMI* body mass index; *SMI* skeletal muscle index; *IPAQ* international physical activity questionnaire; *MET/wk* metabolic equivalent task minutes per week; *MNA-SF* Mini Nutritional Assessment-Short Form; *CCI* Charlson Comorbidity Index

^a^*P* < 0.05 versus Normal, ^b^*P* < 0.05 versus CHD alone, ^c^ *P* < 0.05 versus Sarcopenia alone

**Fig. 2:**
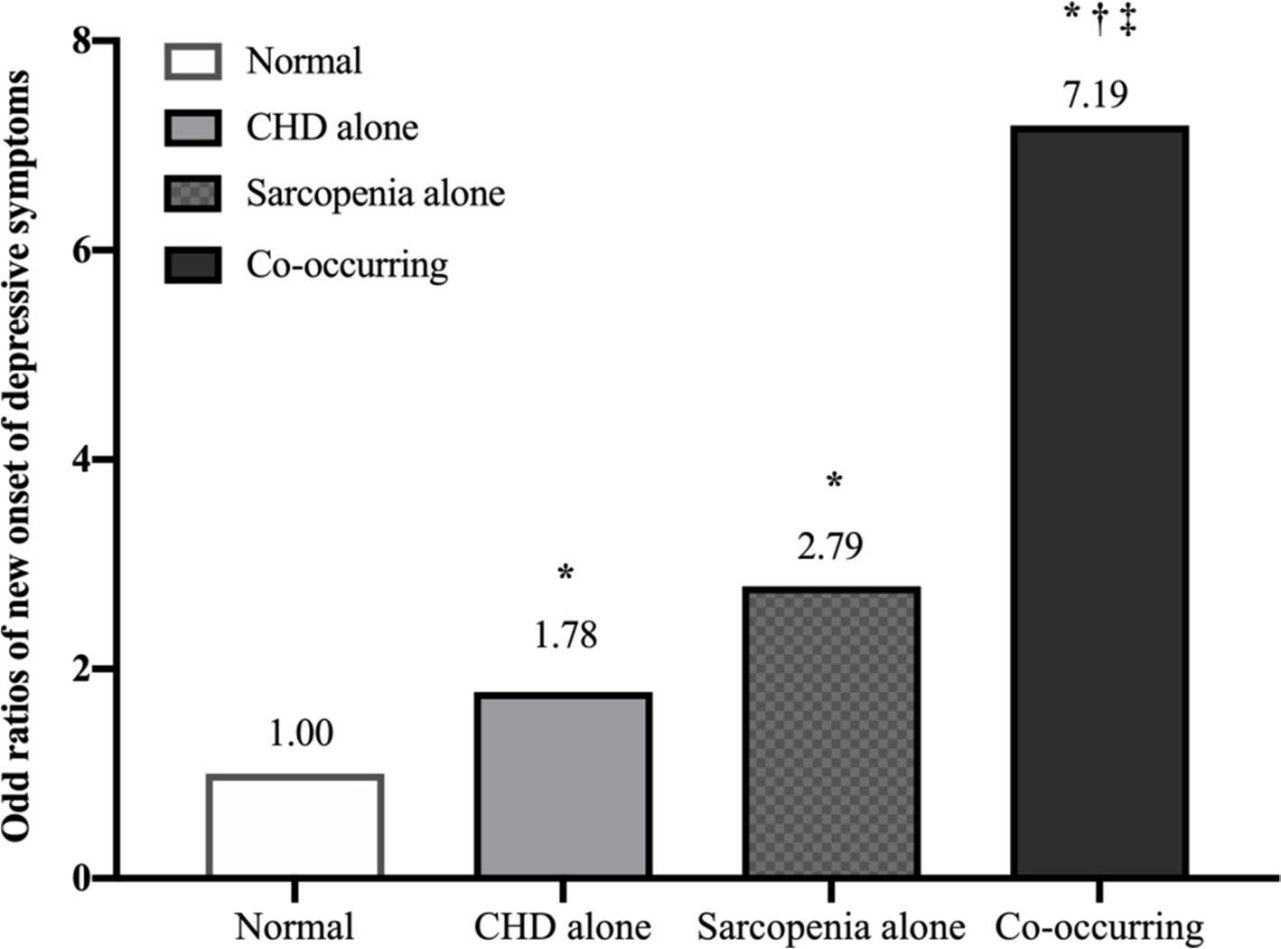
Difference of new onset of depressive symptoms among different groups. *P < 0.05 versus Normal; †*P* < 0.05 versus CHD alone; ‡ *P* < 0.05 versus Sarcopenia alone

Results of the new onset depressive symptoms and logistic regression analysis using the 4 groups are shown in Table 2. New onset depressive symptoms rates of the 4 groups were 8.2% (*n* = 52), 16.7% (*n* = 29), 19.0% (*n* = 12), and 40.0% (*n* = 10) in normal, CHD alone, sarcopenia alone, and co-occurring groups, respectively. Individuals with the new onset depressive symptoms in CHD or sarcopenia group were significantly more than normal group, whereas the co-occurring group owned significantly most elderly with new onset depressive symptoms (*P* < 0.05). After adjustments for potential confounders, the following groups were associated with depressive symptoms incidence from baseline to 1-year follow up, respectively: CHD alone (OR = 1.78, 95% CI = 1.05–3.02), sarcopenia alone (OR = 2.79, 95% CI = 1.26,6.22), and co-occurring (OR = 7.19, 95% CI = 2.75–18.81) groups. No statistical difference was found between CHD alone individuals and sarcopenia alone individuals. However, a significant difference between co-occurring group with CHD alone or sarcopenia alone group or the normal group (*P* < 0.05) (Fig. 2).

**Table 2:** Association of Co-occurring Sarcopenia and CHD with New onset of Depressive Symptoms

Variables	Normal	CHD alone	Sarcopenia alone	Co-occurring	*P*-value
The onset depressive symptoms (%)	52(8.2)	29(16.7)^a^	12(19.0)^a^	10(40.0)^a,b,c^	<0.001
Logistic regression analyses odd ratio (95% CI)					
Unadjusted	1.00(Reference)	2.24(1.38,3.66)^a^	2.64(1.32,5.26)^a^	7.47(3.20,17.47)^a,b,c^	
Adjusted^1^	1.00(Reference)	1.78(1.05,3.02)^a^	2.79(1.26,6.22)^a^	7.19(2.75,18.81)^a,b,c^	

Notes: *CI* confidence interval

1 Adjusted for age, sex, BMI, widowed, education, falling history, drinking, MNA-SF, sleep duration, sleep quality, hypotensive drugs, hypoglycemic drugs, cardiovascular drugs, sleep drugs and CCI

^a^*P* < 0.05 versus Normal, ^b^*P* < 0.05 versus CHD alone, ^c^ *P* < 0.05 versus Sarcopenia alone

## Discussion

To the best of our knowledge, this is the first prospective study to clarify the combined effects of CHD and sarcopenia on new-onset depressive symptoms. In addition, after adjusting for potential confounders, CHD and sarcopenia were found to be independent risk factors of new-onset depressive symptoms. Co-occurring CHD and sarcopenia presented the highest risk of incidence of depressive symptoms in the study population.

In our study, the association between sarcopenia and depression has been discussed deeply in our previous studies [12]. The most compelling finding was that sarcopenia combined with CHD had a further additive predictive value in discriminating older adults at high risk of new-onset depressive symptoms. Previous studies have reported that sarcopenia is closely related to a higher prevalence of established CHD. The presence of sarcopenia is also a contributing factor in poor cardiopulmonary function in patients with CHD [13–15]. Kim et al. [24] believe that the existence of sarcopenia increases the morbidity and mortality of cardiovascular diseases in the elderly. In patients with sarcopenia, the endocrine function of muscle cells is weakened, and muscle cells exert their endocrine functions by secreting cytokines that are beneficial to the cardiovascular system. Both a decrease in the amount of muscle cells and a decline in their endocrine function in patients with sarcopenia could have contributed to the poor clinical outcomes [25]. Behavioural, genetic and inflammatory mechanisms, as well as changes in hormone levels, decreased physical activity, insulin resistance that could explain the presence of sarcopenia and coronary heart disease are linked to depressive symptoms. On the other hand, polyunsaturated omega-3 free fatty acid deficiency, hypothalamic-pituitary-adrenal axis and autonomic mechanisms are also possible link pathways [26].

Another possible underlying mechanism to explain our results is that sarcopenia and CHD form a vicious cycle. Previous research suggested that low muscle mass and muscle strength are associated with risk of atherosclerosis and endothelial dysfunction in the elderly [13, 27], which may worsen the progression of CHD. Our additional results demonstrated that poor physical performance (gait speed <0.8 m/s was associated with new onset of CHD (adjusted OR = 2.08, 95%CI = 1.08–4.00). At the same time, CHD can lead to a decrease in gait speed, reducing people’s willingness to attempt tasks that they are otherwise capable of performing [28]. Subsequently, this decrease in physical performance and increase in restriction might lead to decline of muscle mass or strength, eventually leading to sarcopenia. Based on these previously discussed studies and our current findings, sarcopenia and CHD in older adults appears to have an interaction effect on new-onset depressive symptoms. However, the specific mechanism is still unclear and needs to be confirmed further studies involving larger populations and with longer follow-up durations.

Major differences in body composition, risk of CHD and risk of depression are known to exist between the sexes. In this study, however, we did not explore stratification by gender because of our small sample size. If stratified by gender, co-occurring groups were associated with depressive symptoms incidence from baseline to 1-year follow up in both men and women (men: OR = 18.58, 95% CI = 2.26–152.65; women: OR = 8.51, 95% CI = 2.61–27.74). The man group had a larger confidence interval because there were fewer people in the co-occurring group (*n* = 5), which would limit the reliability of the statistical analysis. However, we can find that the results are statistically significant for both men and women, and the deficiency is that the sample size is small. Therefore, in our statistical model, the final results were adjusted for gender and not stratified by sex in the study. This is consistent with several previous studies, which explored the sarcopenia with depression or CHD without grouping studies by gender [12]. In the future, we need to further expand the sample size to explore whether there are gender differences between sarcopenia, CHD and depression.

This study suggests that older adults with CHD and sarcopenia should be identified early and targeted, so that further mental deterioration and other adverse health outcomes can be prevented. In particular, the American Heart Association (AHA) showed that depression was a risk factor for adverse medical outcomes in patients with CHD [29]. Through this research, we have found the need to focus on physical function. In addition to exercise and nutrition to improve physical function and relieve depression, recent study shows that new drugs can significantly improve depressive symptoms in patients with heart disease [30]. Therefore, much greater attention must be paid to mental health during cardiac rehabilitation and multifaceted interventions require to be considered.

### Strengths and limitations

The strength of this study is that it is the first study to report that CHD with sarcopenia is a stronger risk factor for incidence of depressive symptoms. Moreover, findings may provide new insights for cardiac rehabilitation in older adults. Despite extensive efforts to curb study limitations, some limitations did still exist. First, the present study didn’t describe the severity of CHD or its treatment in sarcopenia patients with CHD. In addition, our participants were relatively healthy, we didn’t include participants who were unable to participate in the free annual national physical examination (eg, those bedridden or with serious disease). Given these reasons, the population studied may not be comprehensive enough. As a result, we may have underestimated the prevalence of sarcopenia, CHD or depressive symptoms. Thirdly, sarcopenia is a major risk factor for frailty, and frailty also has links with depressive symptoms and CHD. We need to acknowledge that the failure to assess frailty is a limitation of this study and we may pay more attention to the older adults with frailty in the future. Fourthly, this study sample was enrolled from a free physical examination program, and the sample size is small, rather than a representative sample. Sarcopenic obesity may act together to increase their effect on metabolic disorders, cardiovascular and mortality [31], while the small number of sample limited our research on sarcopenic obesity. Furthermore, the sample sizes limited our exploration of gender stratification. In the future, when our sample size expands, we will further stratify by gender to explore the correlation between sarcopenic obesity, CHD and depression. Lastly, the follow-up period was short. In future research, we plan to enlarge sample sizes and extend the time for follow-up, to increase the ability to evaluate relationships.

## Conclusions

In conclusion, we have showed the distinct and synergistic effects of sarcopenia and CHD on the risk of new-onset depressive symptoms among community-dwelling older adults in a longitudinal study design, after adjusting for confounding factors. To manage and prevent poor outcomes in older adults, physical, nutritional and psychological assessment and interventions should be implemented to allow patients to maintain functional independence and extend healthy life span expectancy.

## Data Availability

The datasets used and/or analyzed during the current study are available from the corresponding author on reasonable request.

## Abbreviations

CHD: Coronary heart disease
BMI: Body mass index
SMI: Skeletal muscle index
AWGS: Working Group for Sarcopenia
GDS: Geriatric Depression Scale
MNA-SF: Mini Nutritional Assessment-Short Form
IPAQ: International Physical Activity Questionnaire
MET/wk.: Metabolic equivalent task minutes per week
CCI: Charlson Comorbidity Index
PCI: Percutaneous coronary intervention
CABG: Coronary artery bypass graft
AHA: American Heart Association
